# Poly[1-ethyl-3-methyl­imidazolium [tri-μ-chlorido-chromate(II)]]

**DOI:** 10.1107/S1600536809002281

**Published:** 2009-01-28

**Authors:** James J. Danford, Atta M. Arif, Lisa M. Berreau

**Affiliations:** a Department of Chemistry and Biochemistry, Utah State University, 0300 Old Main Hill, Logan, UT 84322-0300, USA; b Department of Chemistry, University of Utah, Salt Lake City, UT 84112-0850, USA

## Abstract

The title compound, {(C_6_H_11_N_2_)[CrCl_3_]}_*n*_, was generated *via* mixing of the ionic liquid 1-ethyl-3-methyl­imidazolium chloride with CrCl_2_ in ethanol. Crystals were obtained by a diffusion method. In the crystal structure, the anion forms one-dimensional chains of chloride-bridged Jahn–Teller distorted chromium(II) centers extending along the [100] direction. The imidazolium cations are positioned between these chains.

## Related literature

For reference to this compound as a possible catalyst for the conversion of glucose to 5-hydroxy­methyl­furfural (HMF), see: Zhao *et al.* (2007 ▶). For the synthesis of the ammonium and tetra­methyl­ammonium analogs [N*R*_4_][CrCl_3_] (*R* = H, CH_3_), see Hardt & Streit (1970 ▶). For the crystal structures of [*M*][CrCl_3_], see: Bellitto *et al.* (1984 ▶) [*M* = N(CH_3_)_4_]; McPherson *et al.* (1972 ▶) (*M* = Cs); Crama *et al.* (1978 ▶) (*M* = Rb, Cs); Crama *et al.* (1979 ▶) (*M* = Rb); Crama & Zandbergen (1981 ▶) (*M* = Cs).
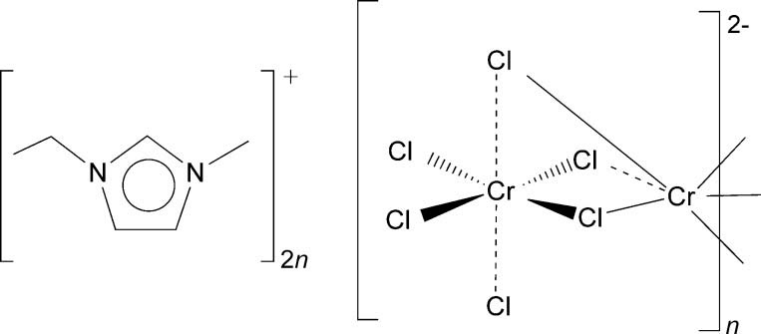


## Experimental

### 

#### Crystal data

(C_6_H_11_N_2_)[CrCl_3_]
*M* *_r_* = 269.52
Monoclinic, 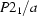

*a* = 6.66150 (10) Å
*b* = 16.4317 (4) Å
*c* = 9.5258 (2) Å
β = 95.6881 (14)°
*V* = 1037.56 (4) Å^3^
*Z* = 4
Mo *K*α radiation
μ = 1.82 mm^−1^
*T* = 150 (1) K
0.25 × 0.20 × 0.15 mm

#### Data collection

Nonius KappaCCD diffractometer
Absorption correction: multi-scan [*DENZO-SMN* (Otwinowski & Minor, 1997 ▶) with scaling algorithm from Fox & Holmes (1966 ▶)] *T*_min_ = 0.659, *T*_max_ = 0.772
4056 measured reflections
2384 independent reflections
2082 reflections with *I* > 2σ(*I*)
*R*_int_ = 0.018

#### Refinement

*R*[*F* ^2^ > 2σ(*F* ^2^)] = 0.026
*wR*(*F* ^2^) = 0.064
*S* = 1.08
2384 reflections
154 parameters
All H-atom parameters refined
Δρ_max_ = 0.42 e Å^−3^
Δρ_min_ = −0.48 e Å^−3^

### 

Data collection: *COLLECT* (Nonius, 1999 ▶); cell refinement: *DENZO-SMN* (Otwinowski & Minor, 1997 ▶); data reduction: *DENZO-SMN*; program(s) used to solve structure: *SIR97* (Altomare *et al.*, 1999 ▶); program(s) used to refine structure: *SHELXL97* (Sheldrick, 2008 ▶); molecular graphics: *WinGX* (Farrugia, 1999 ▶); software used to prepare material for publication: *CrystalMaker* (Palmer, 2005 ▶).

## Supplementary Material

Crystal structure: contains datablocks I, global. DOI: 10.1107/S1600536809002281/si2146sup1.cif

Structure factors: contains datablocks I. DOI: 10.1107/S1600536809002281/si2146Isup2.hkl

Additional supplementary materials:  crystallographic information; 3D view; checkCIF report

## Figures and Tables

**Table:** 

Cr1—Cl2	2.3876 (5)
Cr1—Cl1	2.3898 (5)
Cr1—Cl3	2.4431 (5)
Cr1—Cl3^i^	2.4476 (5)

**Table:** 

Cl2—Cr1—Cl1	177.976 (19)
Cl2—Cr1—Cl3	87.073 (15)
Cl1—Cr1—Cl3	91.904 (16)
Cl2—Cr1—Cl3^i^	91.906 (16)
Cl1—Cr1—Cl3^i^	89.027 (15)
Cl3—Cr1—Cl3^i^	176.95 (2)
Cr1—Cl3—Cr1^ii^	85.856 (13)

Symmetry codes: (i) 
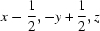
; (ii) 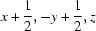.

**Fig. 1.**
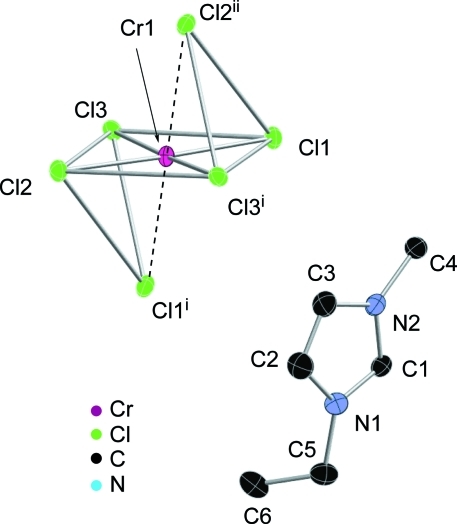
A view of the coordination environment of the chromium center in the trichloridochromate(II) anion and the imidazolium cation with atom labelling for non-hydrogen atoms. Displacement ellipsoids are drawn at the 50% probability level. [Symmetry codes: (i) x - 1/2, -y + 1/2, z; (ii) x + 1/2, -y + 1/2, z.]

**Fig. 2.**
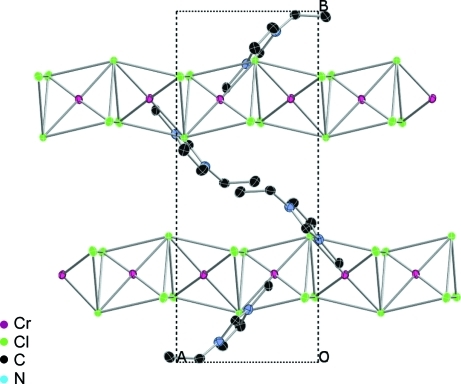
A view of the one-dimensional chain structure of the trichloridochromate(II) anion extending along [100]. Included in the drawing are the four imidazolium cations within the cell. Displacement ellipsoids are drawn at the 50% probability level.

## supplementary crystallographic information

### Comment

Recently it was shown that a solution of CrCl_2 _in the ionic liquid 1-ethyl-3-methylimidazolium chloride ([EMIM]Cl) at 100°C will catalyze the conversion of glucose to 5-hydroxymethylfurfural (HMF) in 70% yield (Zhao *et al.*, 2007). The proposed active catalyst in this system is a compound formulated as [EMIM]CrCl_3_. While alkali metal, ammonium, and tetramethyl ammonium chromium(II) trihalides have been previously reported in the literature (Hardt & Streit, 1970), the title compound is the first structurally characterized imidazolium analog.

The structure consists of infinite linear chains of Jahn–Teller-distorted chromium centers (Fig. 1) bridged by a facial array of chloride ligands (Fig. 2). Each Cr^II^ has four Cr—Cl bonds of σim 2.39–2.45 Å and two longer Cr—Cl interactions (2.87–2.91 Å). The Cr···Cr distance is 3.33 Å. The Cl—Cr—Cl bond angles are in the range of 87–90°. The shortest Cr···Cr distance between chains is 9.19 Å. A number of differences are evident in the structures of [EMIM]CrCl_3_ (collected at 150 (1) K) and the previously reported [N(CH_3_)_4_]CrCl_3_ (collected at room temperature; Bellitto *et al.*, 1984). Specifically, the chromium center in [EMIM]CrCl_3_ has pseudo *D_4_*_h_ site symmetry whereas [N(CH_3_)_4_]CrCl_3_ contains trigonally distorted chromium centers (*C*_3v_ site symmetry) positioned in alternating compressed and elongated face-sharing octahedra. Similar site symmetry to that found in [N(CH_3_)_4_]CrCl_3_ was identified in the room temperature structure of α-CsCrCl_3_, see: McPherson *et al.* (1972) and Crama & Zandbergen (1981). This *C*_3v_ site symmetry is described as resulting from randomly distributed elongation of Cr—Cl bonds along three principal axes of the octahedron.

### Experimental

Under a N_2_ atmosphere, a solution of CrCl_2_ (23 mg, 0.19 mmol) in ethanol (2 ml) was added to solid 1-ethyl-3-methylimidazolium chloride (23 mg, 0.16 mmol). The resulting teal colored solution was stirred at ambient temperature until all of the solid had dissolved. Addition of ethyl acetate (2 ml), followed by diffusion of Et_2_O, produced pale yellow crystals suitable for X-ray analysis.

### Refinement

All H atoms were located and refined isotropically using *SHELXL97* (Sheldrick, 2008).

### Crystal data

**Table:**

(C_6_H_11_N_2_)[CrCl_3_]	*F*(000) = 544
*M**_r_* = 269.52	*D*_x_ = 1.725 Mg m^−^^3^
Monoclinic, *P*2_1_/*a*	Mo *K*α radiation, λ = 0.71073 Å
*a* = 6.6615 (1) Å	Cell parameters from 8584 reflections
*b* = 16.4317 (4) Å	θ = 1.0–27.5°
*c* = 9.5258 (2) Å	µ = 1.82 mm^−^^1^
β = 95.6881 (14)°	*T* = 150 K
*V* = 1037.56 (4) Å^3^	Prism, yellow
*Z* = 4	0.25 × 0.20 × 0.15 mm

### Data collection

**Table:**

Nonius KappaCCD diffractometer	2384 independent reflections
Radiation source: fine-focus sealed tube	2082 reflections with *I* > 2σ(*I*)
graphite	*R*_int_ = 0.018
φ and ω scans	θ_max_ = 27.5°, θ_min_ = 2.5°
Absorption correction: multi-scan [*DENZO-SMN* (Otwinowski & Minor, 1997) with scaling algorithm from Fox & Holmes (1966)]	*h* = −8→8
*T*_min_ = 0.659, *T*_max_ = 0.772	*k* = −20→21
4056 measured reflections	*l* = −12→12

### Refinement

**Table:**

Refinement on *F*^2^	Secondary atom site location: difference Fourier map
Least-squares matrix: full	Hydrogen site location: inferred from neighbouring sites
*R*[*F*^2^ > 2σ(*F*^2^)] = 0.026	All H-atom parameters refined
*wR*(*F*^2^) = 0.064	*w* = 1/[σ^2^(*F*_o_^2^) + (0.0236*P*)^2^ + 0.6211*P*] where *P* = (*F*_o_^2^ + 2*F*_c_^2^)/3
*S* = 1.08	(Δ/σ)_max_ < 0.001
2384 reflections	Δρ_max_ = 0.42 e Å^−^^3^
154 parameters	Δρ_min_ = −0.48 e Å^−^^3^
0 restraints	Extinction correction: *SHELXL97* (Sheldrick, 2008), Fc^*^=kFc[1+0.001xFc^2^λ^3^/sin(2θ)]^-1/4^
Primary atom site location: structure-invariant direct methods	Extinction coefficient: 0.0064 (9)

### Special details

**Table:**

Experimental. The program *DENZO-SMN* (Otwinowski & Minor, 1997) uses a scaling algorithm (Fox & Holmes, 1966) which effectively corrects for absorption effects. High redundancy data were used in the scaling program hence the 'multi-scan' code word was used. No transmission coefficients are available from the program (only scale factors for each frame). The scale factors in the experimental table are calculated from the 'size' command in the *SHELXL97* input file.
Geometry. All e.s.d.'s (except the e.s.d. in the dihedral angle between two l.s. planes) are estimated using the full covariance matrix. The cell e.s.d.'s are taken into account individually in the estimation of e.s.d.'s in distances, angles and torsion angles; correlations between e.s.d.'s in cell parameters are only used when they are defined by crystal symmetry. An approximate (isotropic) treatment of cell e.s.d.'s is used for estimating e.s.d.'s involving l.s. planes.
Refinement. Refinement of *F*^2^ against ALL reflections. The weighted *R*-factor *wR* and goodness of fit *S* are based on *F*^2^, conventional *R*-factors *R* are based on *F*, with *F* set to zero for negative *F*^2^. The threshold expression of *F*^2^ > σ(*F*^2^) is used only for calculating *R*-factors(gt) *etc*. and is not relevant to the choice of reflections for refinement. *R*-factors based on *F*^2^ are statistically about twice as large as those based on *F*, and *R*- factors based on ALL data will be even larger.

### Fractional atomic coordinates and isotropic or equivalent isotropic displacement parameters (Å^2^)

**Table:**

	*x*	*y*	*z*	*U*_iso_*/*U*_eq_	
Cr1	0.30848 (4)	0.251150 (16)	0.79201 (3)	0.01432 (10)	
Cl1	0.09238 (6)	0.18418 (3)	0.61278 (4)	0.01959 (12)	
Cl2	0.52336 (6)	0.31399 (3)	0.97636 (4)	0.01856 (12)	
Cl3	0.55581 (5)	0.14110 (3)	0.79810 (4)	0.01695 (12)	
N1	0.7020 (2)	0.05812 (10)	0.24223 (15)	0.0209 (3)	
N2	0.4931 (2)	0.14965 (9)	0.30051 (15)	0.0191 (3)	
C1	0.5869 (3)	0.11968 (12)	0.19414 (18)	0.0198 (4)	
C2	0.6805 (3)	0.04791 (13)	0.3837 (2)	0.0301 (4)	
C3	0.5517 (3)	0.10515 (13)	0.4202 (2)	0.0281 (4)	
C4	0.3515 (3)	0.21791 (13)	0.2924 (2)	0.0243 (4)	
C5	0.8379 (3)	0.01037 (13)	0.1611 (2)	0.0269 (4)	
C6	1.0520 (3)	0.01492 (15)	0.2275 (3)	0.0339 (5)	
H1	0.574 (3)	0.1415 (13)	0.104 (2)	0.022 (5)*	
H2	0.748 (4)	0.0075 (16)	0.435 (3)	0.043 (7)*	
H3	0.508 (4)	0.1180 (16)	0.506 (3)	0.044 (7)*	
H4A	0.350 (5)	0.2419 (19)	0.206 (4)	0.071 (10)*	
H4B	0.236 (5)	0.1996 (19)	0.309 (3)	0.068 (9)*	
H4C	0.384 (4)	0.2545 (18)	0.356 (3)	0.059 (9)*	
H5A	0.829 (4)	0.0344 (15)	0.067 (3)	0.042 (7)*	
H5B	0.787 (4)	−0.0452 (16)	0.156 (2)	0.040 (6)*	
H6A	1.142 (4)	−0.0168 (17)	0.176 (3)	0.047 (7)*	
H6B	1.061 (3)	−0.0057 (16)	0.319 (3)	0.040 (7)*	
H6C	1.099 (4)	0.0705 (19)	0.240 (3)	0.059 (8)*	

### Atomic displacement parameters (Å^2^)

**Table:**

	*U*^11^	*U*^22^	*U*^33^	*U*^12^	*U*^13^	*U*^23^
Cr1	0.01154 (15)	0.01684 (18)	0.01412 (15)	0.00097 (10)	−0.00098 (10)	−0.00138 (10)
Cl1	0.0171 (2)	0.0254 (3)	0.0157 (2)	−0.00134 (16)	−0.00144 (15)	−0.00269 (16)
Cl2	0.0179 (2)	0.0211 (2)	0.0160 (2)	−0.00030 (15)	−0.00131 (15)	−0.00306 (16)
Cl3	0.0136 (2)	0.0164 (2)	0.0207 (2)	−0.00014 (14)	0.00140 (15)	−0.00127 (15)
N1	0.0225 (7)	0.0204 (8)	0.0203 (7)	−0.0013 (6)	0.0042 (6)	0.0004 (6)
N2	0.0186 (7)	0.0226 (8)	0.0161 (7)	−0.0017 (6)	0.0011 (5)	−0.0013 (6)
C1	0.0210 (8)	0.0225 (9)	0.0161 (8)	−0.0027 (7)	0.0021 (7)	−0.0006 (7)
C2	0.0343 (10)	0.0329 (12)	0.0234 (9)	0.0053 (9)	0.0043 (8)	0.0090 (9)
C3	0.0303 (10)	0.0373 (12)	0.0172 (9)	0.0022 (9)	0.0043 (7)	0.0039 (8)
C4	0.0195 (9)	0.0278 (11)	0.0256 (10)	0.0013 (8)	0.0027 (7)	−0.0044 (9)
C5	0.0284 (10)	0.0215 (10)	0.0321 (10)	−0.0001 (8)	0.0087 (8)	−0.0030 (8)
C6	0.0277 (11)	0.0314 (13)	0.0434 (13)	0.0049 (9)	0.0072 (9)	0.0024 (10)

### Geometric parameters (Å, °)

**Table:**

Cr1—Cl2	2.3876 (5)	C2—H2	0.91 (3)
Cr1—Cl1	2.3898 (5)	C3—H3	0.91 (3)
Cr1—Cl3	2.4431 (5)	C4—H4A	0.91 (3)
Cr1—Cl3^i^	2.4476 (5)	C4—H4B	0.86 (3)
N1—C1	1.323 (2)	C4—H4C	0.86 (3)
N1—C2	1.380 (2)	C5—C6	1.503 (3)
N1—C5	1.473 (2)	C5—H5A	0.97 (2)
N2—C1	1.336 (2)	C5—H5B	0.97 (3)
N2—C3	1.378 (2)	C6—H6A	0.96 (3)
N2—C4	1.463 (3)	C6—H6B	0.93 (3)
C1—H1	0.93 (2)	C6—H6C	0.97 (3)
C2—C3	1.342 (3)		
			
Cl2—Cr1—Cl1	177.976 (19)	C2—C3—H3	131.2 (17)
Cl2—Cr1—Cl3	87.073 (15)	N2—C3—H3	121.7 (17)
Cl1—Cr1—Cl3	91.904 (16)	N2—C4—H4A	109 (2)
Cl2—Cr1—Cl3^i^	91.906 (16)	N2—C4—H4B	108 (2)
Cl1—Cr1—Cl3^i^	89.027 (15)	H4A—C4—H4B	113 (3)
Cl3—Cr1—Cl3^i^	176.95 (2)	N2—C4—H4C	112 (2)
Cr1—Cl3—Cr1^ii^	85.856 (13)	H4A—C4—H4C	108 (3)
C1—N1—C2	108.55 (16)	H4B—C4—H4C	106 (3)
C1—N1—C5	126.20 (16)	N1—C5—C6	111.08 (17)
C2—N1—C5	125.20 (17)	N1—C5—H5A	106.3 (14)
C1—N2—C3	108.45 (16)	C6—C5—H5A	109.7 (14)
C1—N2—C4	126.18 (16)	N1—C5—H5B	107.4 (14)
C3—N2—C4	125.37 (15)	C6—C5—H5B	112.2 (14)
N1—C1—N2	108.52 (15)	H5A—C5—H5B	110 (2)
N1—C1—H1	127.8 (13)	C5—C6—H6A	111.9 (15)
N2—C1—H1	123.6 (13)	C5—C6—H6B	110.2 (15)
C3—C2—N1	107.38 (18)	H6A—C6—H6B	107 (2)
C3—C2—H2	131.7 (16)	C5—C6—H6C	112.4 (17)
N1—C2—H2	121.0 (16)	H6A—C6—H6C	111 (2)
C2—C3—N2	107.08 (16)	H6B—C6—H6C	104 (2)
			
Cl2—Cr1—Cl3—Cr1^ii^	−48.298 (16)	C5—N1—C2—C3	176.88 (18)
Cl1—Cr1—Cl3—Cr1^ii^	133.450 (13)	N1—C2—C3—N2	0.6 (2)
C2—N1—C1—N2	0.4 (2)	C1—N2—C3—C2	−0.4 (2)
C5—N1—C1—N2	−177.10 (16)	C4—N2—C3—C2	179.39 (18)
C3—N2—C1—N1	0.0 (2)	C1—N1—C5—C6	121.0 (2)
C4—N2—C1—N1	−179.80 (17)	C2—N1—C5—C6	−56.1 (3)
C1—N1—C2—C3	−0.7 (2)		

Symmetry codes: (i) *x*−1/2, −*y*+1/2, *z*; (ii) *x*+1/2, −*y*+1/2, *z*.
